# Breathing Rate Estimation From the Electrocardiogram and Photoplethysmogram: A Review

**DOI:** 10.1109/RBME.2017.2763681

**Published:** 2017-10-24

**Authors:** Peter H. Charlton, Drew A. Birrenkott, Timothy Bonnici, Marco A. F. Pimentel, Alistair E. W. Johnson, Jordi Alastruey, Lionel Tarassenko, Peter J. Watkinson, Richard Beale, David A. Clifton

**Affiliations:** Department of Biomedical Engineering, King’s College London, London SE1 7EH, U.K., and also with the Department of Engineering Science, University of Oxford, Oxford OX3 7DQ, U.K; Department of Engineering Science, University of Oxford, Oxford OX3 7DQ, U.K; Nuffield Department of Medicine, University of Oxford, Oxford OX3 9DU, U.K., and also with the Department of Asthma, Allergy, and Lung Biology, King’s College London, London SE1 7EH, U.K; Department of Engineering Science, University of Oxford, Oxford OX3 7DQ, U.K; Laboratory for Computational Physiology, Massachusetts Institute of Technology, Cambridge, MA 02139 USA; Department of Biomedical Engineering, King’s College London, London SE1 7EH, U.K; Department of Engineering Science, University of Oxford, Oxford OX3 7DQ, U.K; Kadoorie Centre for Critical Care Research and Education, Oxford University Hospitals NHS Foundation Trust, Oxford OX3 9DU, U.K.; Department of Asthma, Allergy and Lung Biology, King’s College London, London SE1 7EH, U.K.; Department of Engineering Science, University of Oxford, Oxford OX3 7DQ, U.K

**Keywords:** Biomedical signal processing, breathing rate (BR), electrocardiogram (ECG), photoplethysmogram (PPG), respiratory rate

## Abstract

Breathing rate (BR) is a key physiological parameter used in a range of clinical settings. Despite its diagnostic and prognostic value, it is still widely measured by counting breaths manually. A plethora of algorithms have been proposed to estimate BR from the electrocardiogram (ECG) and pulse oximetry (photoplethysmogram, PPG) signals. These BR algorithms provide opportunity for automated, electronic, and unobtrusive measurement of BR in both healthcare and fitness monitoring. This paper presents a review of the literature on BR estimation from the ECG and PPG. First, the structure of BR algorithms and the mathematical techniques used at each stage are described. Second, the experimental methodologies that have been used to assess the performance of BR algorithms are reviewed, and a methodological framework for the assessment of BR algorithms is presented. Third, we outline the most pressing directions for future research, including the steps required to use BR algorithms in wearable sensors, remote video monitoring, and clinical practice.

## I Introduction

BREATHING rate (BR) is a key physiological parameter used in a range of clinical settings for identification of abnormalities. Despite this, it is still widely measured by counting breaths manually. This approach is both labor intensive and unsuitable for use in unobtrusive monitoring devices for early detection of deteriorations. Recently, a plethora of algorithms have been proposed to estimate BR from the electrocardiogram (ECG) and pulse oximetry (photoplethysmogram, PPG) signals. Both the ECG and PPG are commonly acquired during clinical assessment, and also by many wearable sensors in healthcare and fitness monitoring. Therefore, BR algorithms could provide automated, electronic BR measurements without the need for additional sensors.

The aims of this paper are: to provide a comprehensive review of the literature on BR estimation from the ECG and PPG; to present a methodological framework for the assessment of BR algorithms; and to highlight the most pressing directions for future research. The background to the problem is summarized in the remainder of this section. In Section II, we present the methodology and results of a review of the literature on the topic. The BR algorithms reported in the literature are reviewed in Section III. Section IV-A provides a critical review of the experimental methodologies used previously to assess the performance of BR algorithms. In Section IV-B, we present a methodological framework for assessment of BR algorithms. Finally, in Section V, we highlight the most pressing directions for future research. This review builds on the work presented in [1].

### A Importance of BR

BR is a valuable diagnostic and prognostic marker of health (also known as respiratory rate). In hospital healthcare, it is a highly sensitive marker of acute deterioration [2]. For instance, elevated BR is a predictor of cardiac arrest [3] and in-hospital mortality [4], and can indicate respiratory dysfunction [5]. Consequently, BR is measured every 4–6 h in acutely ill hospital patients [6]. BR is also used in emergency department screening [7]. In primary care, BR is used in the identification of pneumonia [8], [9] and sepsis [10], [11], and as a marker of hypercarbia [12] and pulmonary embolism [13], [14]. However, BR is usually measured by manually counting chest wall movements (outside of intensive care). This process is time consuming, inaccurate [15], [16], and poorly carried out [12], [17]. Furthermore, BR monitoring is not widely incorporated into wearable sensors such as fitness devices [18]. Therefore, there is potentially an important role for an unobtrusive, electronic method for measuring BR, such as the estimation of BR from the ECG or PPG.

### B ECG and PPG

The ECG and PPG are easily and widely acquired by non-invasive sensors in both healthcare and consumer electronics devices, making them suitable candidates for BR measurement in a range of settings.

The ECG is a measure of the electrical current generated by the action potentials in the myocardium (heart muscle) each heartbeat. It is acquired by measuring the voltage difference between two points on the body surface over time caused by this current [19]. The ECG can be measured using low-cost circuitry and electrodes (typically applied to the thorax) [20]. Static monitors are used to obtain single ECG measurements during screening for heart disorders and for continuous monitoring in critical care units. ECG monitoring is also incorporated into wearable sensors for use with ambulatory patients to identify changes in heart rate (HR) and rhythm [21] and in personal fitness devices.

The PPG is a measure of changes in blood volume over time in a bed of tissue [22]. It is measured by applying a sensor to the skin, or by noncontact imaging of a region of the skin using a camera [23]. A tissue bed is illuminated by either a supplementary light source (such as an LED) [24] or ambient light [25]. The intensity of light transmitted through or reflected from the bed is then measured by a photodetector [26]. Contact PPG measurements are commonly performed at peripheral sites (such as the finger or ear) using a low-cost pulse oximeter probe, which can be quickly attached [10]. Noncontact measurements are performed by measuring the light reflected from areas of exposed skin, such as the face or hand [23], [27]. Smartphones and tablets can also be used to acquire contact and noncontact PPG signals [28], [29]. The PPG is routinely measured in a wide range of clinical settings to obtain peripheral arterial blood oxygen saturation (SpO_2_) and pulse rate measurements.

It is continuously monitored in critically ill patients and can be monitored in ambulatory patients using wearable sensors [30]. In addition, the PPG is used for continuous HR monitoring in fitness devices [31]. Further applications of the PPG are being developed, including blood perfusion assessment and pulse transit time measurement. These use PPG signals obtained simultaneously at multiple sites from a single noncontact imaging PPG [23].

### C Respiratory Modulation of the ECG and PPG

**Fig. 1:**
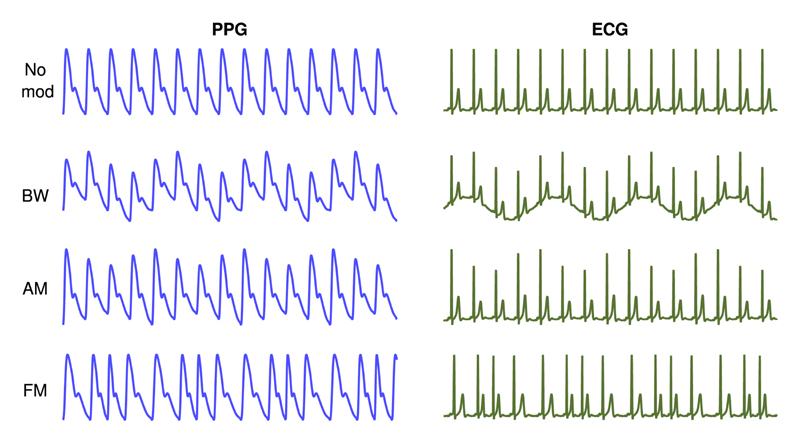
ECG and PPG are subject to three respiratory modulations: baseline wander (BW), amplitude modulation (AM), and frequency modulation (FM). Source: [33] (CC BY-NC 4.0: http://creativecommons.org/licenses/by-nc/4.0/).

It is widely reported that the ECG and PPG both exhibit three respiratory modulations as illustrated in Fig. 1: baseline wander (BW), amplitude modulation (AM), and frequency modulation (FM) [8], [13], [18], [32]. BR algorithms estimate BR by analyzing one or more of these modulations [8], [31].

The physiological mechanisms that cause respiratory modulations can be summarized as follows [34]. BW and AM of the ECG are caused by changes in the orientation of the heart’s electrical axis relative to the electrodes and changes in thoracic impedance [35]. BW of the PPG is due to changes in tissue blood volume caused by: changes in intrathoracic pressure transmitted through the arterial tree; and vasoconstriction of arteries during inhalation transferring blood to the veins [36]. AM of the PPG is caused by reduced stroke volume during inhalation due to changes in intrathoracic pressure, reducing pulse amplitude [37]. FM is the manifestation of the spontaneous increase in HR during inspiration, and decreases during exhalation, known as respiratory sinus arrhythmia (RSA) [38]. RSA is caused by at least three mechanisms [34], which are as follows:

1) changes in intrathoracic pressure during inhalation stretch the sinoatrial node, increasing HR;
2) increased vagal outflow during exhalation reduces HR; and
3) reduced intrathoracic pressure during inhalation decreases left ventricular stroke volume, causing a baroreflex-mediated increase in HR [39].

The strengths of each modulation may differ between subjects and between patient groups [13]. Indeed, large intersubject variations have been observed [34], [40]. Furthermore, particular modulations may be diminished in certain groups, such as FM in elderly subjects [34]. Consequently, many BR algorithms analyze multiple modulations, providing improved performance [8], [18].

## II Search Strategy and Results

A review of the literature was performed to identify publications describing BR algorithms for use with the ECG or PPG. Publications were identified through manual searches and searches of online databases (Google Scholar, IEEE *Xplore*, PubMed, Science Direct, and Scopus). Additional details of the search strategy are provided in Section S1 (Supplimentary Material), allowing the search to be reproduced and updated.

A total of 196 publications describing BR algorithms were identified, which form the basis for this review [8], [10], [13], [18], [24], [28], [29], [31]–[33], [41]–[226]. The earliest publication was in 1971 [211], and only nine more were published between then and 1998. Since 1999, the rate of publication has risen steadily to the present rate of approximately 20 publications per year (see Fig. S2 [Supplimentary Material]). This demonstrates the increasing interest in BR algorithms and the importance placed upon the topic. Approximately half of the publications (101, 51.5%) were presented at conferences. The remainder were journal articles (88, 44.9%), theses (5, 2.6%), or book chapters (2, 1.0%).

## III BR Algorithms

**Fig. 2:**
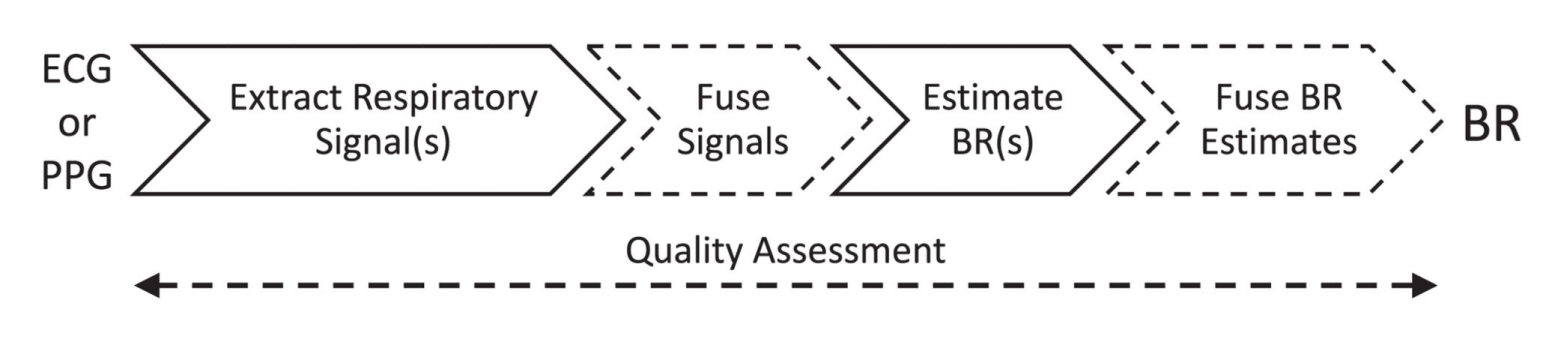
Stages of a BR algorithm. Dashed stages are optional.

BR algorithms can be considered to consist of up to five stages, as illustrated in Fig. 2.

The role of each stage is as follows.

1) *Extract Respiratory Signal(s):* consists of extracting one or more signals dominated by respiratory modulation.
2) *Fusion of Respiratory Signals:* multiple respiratory signals can be fused to give one respiratory signal (optional).
3) *Estimate BR(s):* consists of estimating a BR from a window of a respiratory signal.
4) *Fuse BR(s):* multiple BR estimates can be fused to obtain one final estimate (optional).
5) *Quality Assessment:* used to reject or mitigate against imprecise estimates (optional).

The mathematical techniques that have been used at each stage are summarized in this section. Some of the content in this section has been adapted from [18] and [34] (CC BY 3.0: http://creativecommons.org/licenses/by/3.0/) and [1] (CC BY 4.0: http://creativecommons.org/licenses/by/4.0/).

### A Extraction of Respiratory Signals

**Fig. 3:**
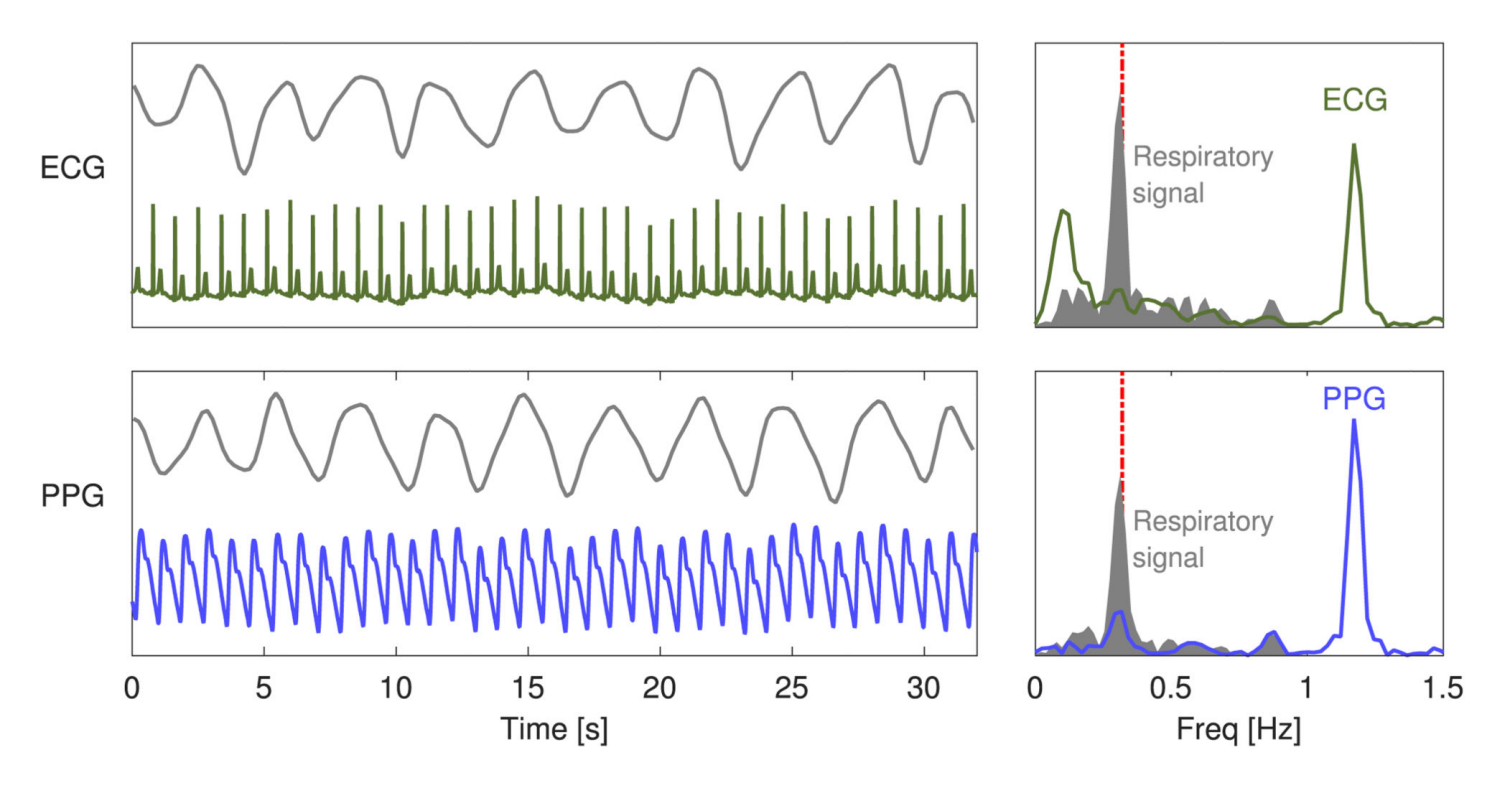
Extraction of exemplary respiratory signals: ECG (upper plot) and PPG (lower plot) signals and extracted respiratory signals (grey) are shown on the left. The corresponding frequency spectra are shown on the right. The frequency spectra of the raw ECG and PPG signals are dominated by cardiac frequency content at 1.2 Hz. In contrast, the extracted respiratory signals are dominated by respiration at 0.3 Hz, which is approximately the BR provided by a reference respiratory signal (shown by the dashed line).

ECG and PPG signals are primarily cardiac in origin, with secondary respiratory modulations of much lower magnitudes. Therefore, the first stage of a BR algorithm is the extraction of a signal dominated by respiratory modulation from which BR can be more easily estimated, as demonstrated in Fig. 3.

Techniques for extraction of a respiratory signal fall into two categories: filter based or feature based [13]. Filter-based techniques consist of filtering the raw signal to attenuate nonrespiratory frequency components (e.g., bandpass filtering the PPG to extract a respiratory signal exhibiting BW). Feature-based techniques consist of extracting beat-by-beat feature measurements (e.g., the amplitude of each QRS complex). The individual processing steps used for extracting a respiratory signal are now described.

#### 1) Elimination of Very Low Frequencies

The first step is the elimination of very low frequency (VLF) components of the PPG and ECG, i.e., those at subrespiratory frequencies. VLFs have been eliminated through high-pass filtering using: a median filter [74], [174], [179]; subtraction of a baseline trend calculated using a linear or polynomial fit [10], [148]; or measurements of the baseline at a specific point in the cardiac cycle (e.g., shortly before the QRS complex [147], or at midpoints between successive R waves [47] in the ECG). A cutoff frequency between 0.03 and 0.05 Hz is typically chosen [148], [196], [197], [205]. This step is beneficial regardless of whether a filter- or feature-based technique is being used, unless VLFs are removed during data acquisition, for instance by some commercial monitors [26].

#### 2) Filter-Based Techniques

Filter-based techniques for extraction of a respiratory signal are performed in a single step. Several techniques have been proposed, as listed in Table I.

#### 3) Feature-Based Techniques

**Fig. 4:**
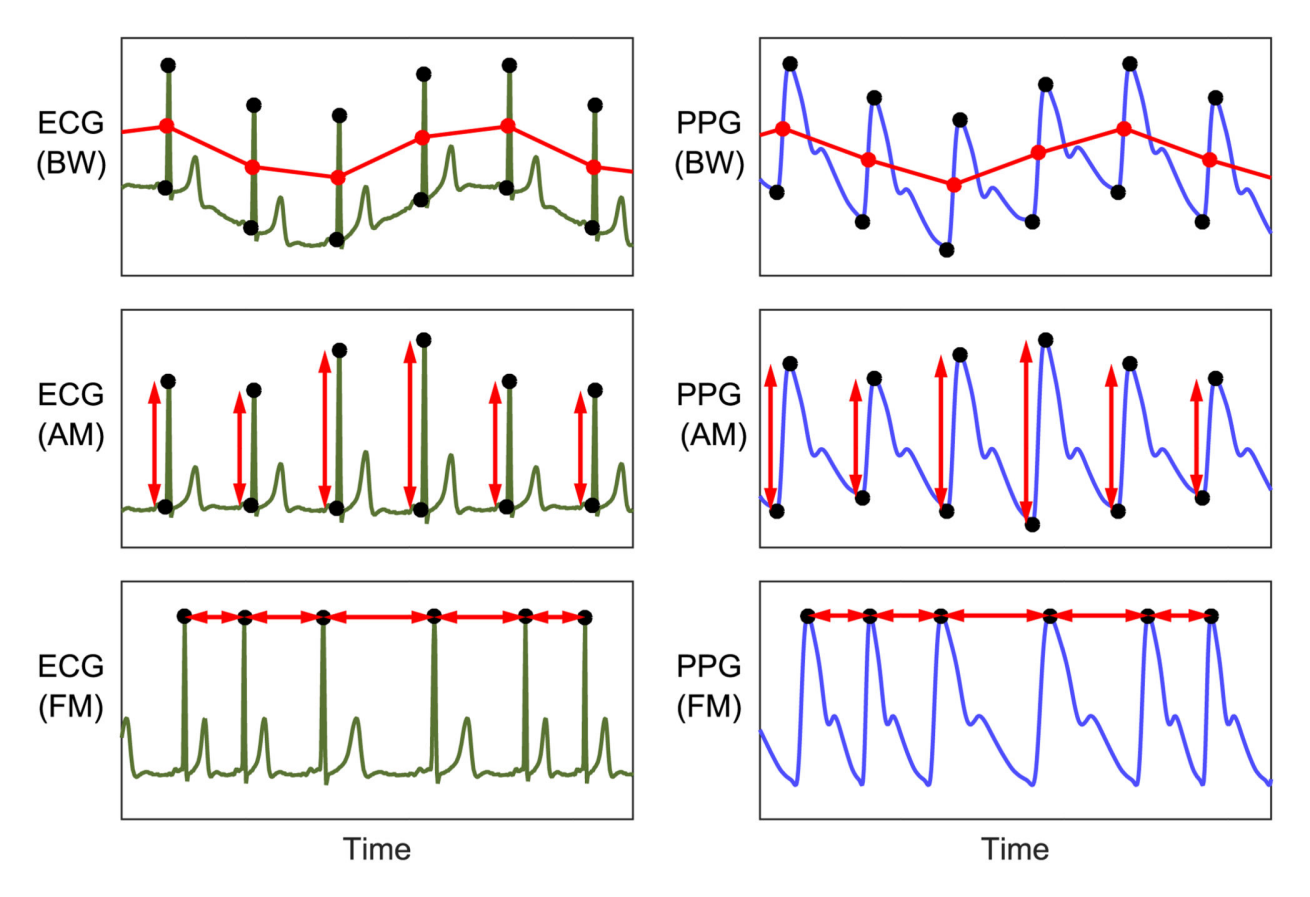
Exemplary feature-based techniques for extraction of respiratory signals from ECG (left) and PPG (right) signals: measurements of baseline wander (BW), amplitude modulation (AM), and frequency modulation (FM) have been extracted for each beat from fiducial points (shown as dots). Adapted from [33] (CC BY-NC 4.0).

Feature-based techniques involve several steps to extract a time series of beat-by-beat features. Examples of the use of feature-based techniques are shown in Fig. 4. The first step is the elimination of very high frequency (VHF) noise by low-pass filtering to improve the accuracy of beat detection and feature measurements. Higher cutoff frequencies are used for the ECG (e.g., 40, 75, or 100 Hz [61], [97], [197]) than the PPG (e.g., 10 or 35 Hz [97], [114]), to preserve the high-frequency content of the QRS complex. In addition, the ECG is particularly susceptible to power-line interference, which may be eliminated using an additional band-stop filter [110]. Commercial monitoring devices typically remove VHFs internally, similar to VLFs [26]. Next, individual beats are detected (see Section S2-A [Supplimentary Material] for details of beat detectors used in the literature). Fiducial points (such as Q- and R-waves, shown as black dots in Fig. 4) are then identified for each beat. These are used to measure a feature that varies with respiration (such as the difference in amplitudes between Q- and R-waves for AM). The fiducial points identified and subsequent feature measurements are specific to the particular feature-based technique being used, as summarized in Table II (additional features are proposed in [40]). Several feature-based techniques use multilead ECG signals [35] or nonstandard leads derived from them [113]. Lastly, the time series of beat-by-beat feature measurements is resampled at a regular sampling frequency of approximately 4–10 Hz. This is usually necessary since signals obtained from beat-by-beat feature measurements are irregularly sampled (once per beat), whereas subsequent processing often requires a regularly sampled signal. Often linear [8], [210] or cubic spline interpolation [110] is used. More complex methods include: Berger’s algorithm, designed for use with an FM signal [228], used in [8] and [101], the integral pulse frequency modulation model, also designed for use with FM signals [229], used in [193]; and the discrete wavelet transform [179].

#### 4) Elimination of Nonrespiratory Frequencies

Nonres-piratory frequencies should be removed from respiratory signals to avoid erroneously identifying spurious frequency content as the BR. Bandpass filtering has been used, with cutoffs at either end of the range of plausible respiratory frequencies [50], [74], [110], [114], [171]. There is no consensus on the optimal range of plausible respiratory frequencies. Furthermore, it may need to be adjusted according to the patient population, particularly for children [230]. Indeed, some BR algorithms use a range that adapts to the HR [85], [128], [159], [160], [209]. As a guideline, Karlen *et al.* used a conservative range of 4–65 breaths per minute (bpm) [8].

### B Fusion of Respiratory Signals

The second stage is the fusion of multiple respiratory signals to provide one respiratory signal from which BR can be estimated. Multiple respiratory signals can be obtained either by extracting multiple signals simultaneously (e.g., by using both the ECG and PPG [44] or by using multiple extraction methods [109]) or by segmenting a respiratory signal into several (often overlapping) windows and treating these as individual signals [51]. Techniques for fusion of multiple respiratory signals are listed in Table III. This stage is optional and is intended to increase the accuracy and robustness of BR estimates [44].

Techniques for fusion of simultaneous respiratory signals result in a single respiratory signal with enhanced respiratory content and reduced spurious frequency content. These techniques, such as spectral averaging, can improve BR algorithm accuracy even beyond that achieved by using the respiratory signal with the strongest respiratory modulation [110], [114]. This is beneficial since the relative strengths of different modulations are often unknown, since it can vary between individuals and with BR [40]. The contribution of spurious frequencies, such as Traube–Hering–Mayer waves at ≈0.1 Hz [132], is reduced since these are unlikely to be manifested consistently across all respiratory signals. Fusion of spectra obtained from short segments of a single respiratory signal reduces variance and increases robustness [110]. This is particularly advantageous during exercise, when there is significant motion artifact [53]. Fusion techniques often incorporate quality assessment that excludes signals from the calculation which exhibit little respiratory modulation [110]. This can prevent a BR from being calculated if there is insufficient respiratory modulation [114], which is appropriate when monitoring BR continuously to detect abnormalities.

### C Estimation of BRs

The third stage of BR algorithms is the estimation of BR. The input to this stage is a window of a respiratory signal and the output is a BR estimate. The techniques used for this stage, listed in Table IV, act in either the time or frequency domain. Time-domain techniques involve detecting individual breaths, followed by calculation of the BR as the mean breath duration. Time-domain techniques have the advantage of not requiring a quasi-stationary BR although they are susceptible to spurious breath detection due to abnormal respiratory signal morphology [18]. Frequency-domain techniques involve identifying the frequency component related to respiration, typically through spectral analysis or identification of the instantaneous dominant frequency. One aspect of using AR frequency-domain techniques is the selection of a model order, detailed in Section 2-B (Supplementary Material). The BR estimation stage may be the last in a BR algorithm. However, two further stages can optionally be used and are now described.

### D Fusion of BRs

Techniques for fusion of multiple BR estimates can be used to improve the robustness of a final BR estimate. Several approaches have been used to fuse simultaneous BR estimates derived from different respiratory signals. First, BRs can be fused by averaging using the mean, median, or mode [8], [64], [176], optionally after exclusion of outliers [64], [113]. The quality of the final estimate can then be assessed from the standard deviation of the individual estimates [8]. Second, BRs can be combined by weighting them according to their variances [13], [226]. Third, a Kalman filter can be used to fuse BRs, which can be weighted according to confidence metrics [128], [163]. Fourth, candidate BRs obtained through AR modeling can be fused using the pole magnitude criterion [169] or the pole ranking criterion [171]. Finally, BRs derived from a single respiratory signal at different time points can be fused using temporal smoothing [114] or particle filtering [122].

### E Quality Assessment

Quality assessment techniques are optional and fall into two categories: signal quality indices (SQIs) and respiratory quality indices (RQIs).

SQIs are used to identify segments of ECG or PPG data of low quality, which are typically rejected based on the assumption that BRs derived from them are likely to be inaccurate [8]. SQIs based on template matching involve constructing a template of average beat morphology and calculation of the correlation between individual beats and the template [231]. A signal segment is deemed to be of high or low quality by comparing the average correlation coefficient for that segment to an empirically determined threshold. Hjorth parameters have also been used, quantifying the strength of an oscillation in a signal [176]. Furthermore, signal quality can be assessed using multiple beat detectors, with agreement between detectors indicating high quality [161]. Beat-by-beat characteristics have also been analyzed to identify low-quality input signals, including beat-to-beat intervals, pulse amplitudes, and clipped pulses [8].

In addition to SQIs, a relatively recent development in the quality assessment of BR algorithms has been the derivation of RQIs [56], [65], [101], [163], [232]. RQIs are used to assess the quality of extracted respiratory signals. RQIs are an important development since the presence or absence of respiratory modulations of the ECG or PPG is independent of the overall quality of those signals and instead varies based on factors such as gender, age, pre-existing health conditions, level of hydration, body position, and the value of the BR itself [40], [163], [233]. Presently, RQIs assess the quality of respiratory signals based on the regularity of breathing peaks and the periodicity of the respiratory waveform. Time- and frequency-domain techniques have been used including: statistical analysis of the variations in the respiratory peaks [65], Hjorth descriptors, Fourier transform, autoregression, and autocorrelation [56], [163]. Because RQIs return a range of values (often between 0 and 1) as opposed to a binary outcome, one of the important considerations in RQI implementation is the compromise between data retention and improved estimation accuracy. Recent results using RQIs to fuse BR estimates from multiple ECG and PPG modulations have shown that RQIs both increase data retention and decrease estimation error compared to existing fusion methods [232]. Further work is required to investigate the performance of RQIs in the presence of diseases that cause irregular or shallow breathing, such as in premature infants at risk of apnoea.

## IV Assessment of BR Algorithms

### A Assessment Methodologies Used in the Literature

A wide range of methodologies were used to assess the performance of BR algorithms in the 196 publications. These are summarized in Table V and critically reviewed in this section. The methods used to obtain Table V are described in Section S3 (Supplimentary Material).

The literature has focused on the development of novel BR algorithms rather than comparisons of existing algorithms. This is shown by approximately half (48.0%) of publications assessing the performance of only one algorithm, without any comparator. Furthermore, only nine publications (4.6%) compared more than ten algorithms. Several issues make it difficult to compare the reported performance of algorithms between different publications: the use of different statistical measures, the use of data from different subject populations, and the lack of standardized implementations of algorithms (with the exception of [227]), to name but a few [18]. Consequently, it is not possible to determine from the literature which algorithms perform best. The RRest Toolbox (http://peterhcharlton.github.io/RRest) has been designed to address these issues [18], [33], [34]. It provides standardized implementations of several algorithms, as well as code to assess their performance using a range of statistics across multiple publicly available datasets. Further comparisons of algorithms would provide equipment designers with much needed evidence to determine which algorithms are most suitable for implementation.

Most algorithms assessed in the literature take either the ECG or PPG as the input signal (used in 50.0% and 57.1% of publications, respectively). Very few publications reported algorithms using both ECG and PPG [44], [81], [138], [145], [170] or pulse transit time [44], [69], [81], [97], [110], [111], [114], [213]. It may be beneficial to use multiple input signals when they are available, such as in wearable sensors [170].

There are pros and cons to the use of shorter and longer window durations with BR algorithms. Most studies used durations of between 30 and 90 s although durations of 5–300 s have been used [184], [216]. Several studies have investigated the impact of window duration on performance [8], [31], [51], [55], [71], [108], [184], [206], [216]. A (nonsignificant) trend toward lower errors at longer window durations has been reported [8], [31] although there is not yet a consensus as to the optimal window length. The optimal length is likely to differ between populations and applications [55]. On one hand, using shorter windows reduces both the time required to measure BR and the computational requirements of BR algorithms [8]. It also increases the likelihood that the BR is stable throughout the window and allows variations in BR to be tracked more accurately, both of which are concerns during exercise [51]. On the other hand, longer windows may improve the accuracy of algorithms and increase the range of detectable BRs [31]. Consequently, a duration of 32 s was chosen as a compromise in [8] and [206].

The datasets used were often not representative of target populations and not publicly available. Datasets were often acquired from young, healthy subjects. Fewer publications used data acquired from elderly adults (25.5%), patients suffering from chronic diseases in the community (11.2%), or acutely ill patients in hospital (7.7%), who are more representative of target patient populations. In addition, few publications used ambulatory data (16.3%). Some publications used data from ventilated patients (16.3%) or subjects breathing in time with a metronome (23.0%). It is not yet clear whether the respiratory mechanics of these subjects can be presumed to be similar to those of spontaneously breathing patients [18]. Consequently, it is not clear whether the performance of BR algorithms reported in these studies is truly indicative of expected performance in target populations. A total of 13 publicly available datasets have been used to assess BR algorithms (see Table VI). However, only two publications have used more than two datasets [57], [111]. The range of available datasets makes it possible to assess algorithms across multiple datasets, which is important as performance may differ significantly between datasets [31].

A range of techniques have been used to acquire reference BRs. Typically, a respiratory signal such as ImP was acquired from which reference BRs were estimated using a bespoke algorithm. Many bespoke algorithms were used although often there was no assessment of the performance of these algorithms. This makes it difficult to know whether errors in BR estimates derived from the ECG and PPG were solely due to poor BR algorithm performance or contributed to by inaccuracies in reference BRs. Notable exceptions are [18], [155], and [184]. In [155], eight methods were used to obtain reference BRs and the final estimate was calculated as the mean of the three estimates closest to the median. In [184], several algorithms for obtaining reference BRs were compared and time-domain breath detection methods were found to be *“the only serious candidates,”* with frequency-domain spectral methods and an autocorrelation method performing poorly. In [18], a time-domain breath detection algorithm was also used and its performance was quantified by comparing the reference BRs provided by the algorithm to those obtained from manual annotations of a subset of the data. An alternative approach is to manually annotate individual breaths in the entire dataset [8], [31]. Regardless of the approach chosen, it is important that reference BRs are accurate for robust assessment of BR algorithms.

A wide range of statistics have been used to assess BR algorithm performance. Statistics were most commonly calculated from the errors between reference and estimated BRs (used in 64.8% of publications), including the mean (absolute) error, root-mean-square error, and the percentage error. The related LOAs method, consisting of the systematic bias and LOAs within which 95% of errors are expected to lie, was used less often (23.5%) even though this has the advantage of quantifying both accuracy and precision [18]. This method is useful because certain applications require greater accuracy (such as identification of pneumonia indicated by BR > 40 bpm [8]), whereas others require greater precision (such as detection of acute changes in BR indicative of deterioration [18]). Statistics indicating the reliability with which individual breaths are detected were used in 9.7% of publications. These included statistics such as sensitivity, specificity, false negative, and false positive rates. Correlation coefficients were used in a minority of publications (13.8%). The wide range of statistics reflects the difficulty of quantifying the performance of algorithms using one single metric.

### B Methodological Framework for Algorithm Assessments

We now present a general methodological framework for assessment of BR algorithms. The reader is referred to [1, Chs. 6–7] for examples of BR algorithm assessments conducted in line with this framework.

#### 1) Purpose of Assessment

It is important to identify the purpose of an algorithm assessment: either exploratory analysis or validation of a BR algorithm. Exploratory analyses are used to determine the performance of a novel algorithm, often in comparison to existing algorithms [31], [90]. They provide evidence to inform the direction of algorithm development and can be used to identify candidate algorithms for validation studies. Validation studies assess BR algorithms to determine whether they are suitable for a particular application [43]. The purpose of the assessment informs the study design.

#### 2) Dataset(s)

The dataset required for an assessment differs according to its purpose. In a validation study, the dataset should be as representative as possible of the intended application, to ensure the results indicate the expected performance. The subject population should be closely matched to the intended users, including: age, level of illness, range of BRs, and type of breathing. Signal acquisition equipment should be similar to that which will be used, considering: transducers, signal fidelity (sampling frequency and resolution), and any signal filtering. The recording setting, including the presence or absence of subject movement, should also be similar. If any publicly available datasets (see Table VI) meet these criteria, then they can be used. Otherwise, a novel dataset should be acquired. The requirements for datasets in exploratory analyses are less stringent. In fact, variation within a dataset can allow a greater range of hypotheses to be tested, such as: multiple heart rhythms [216]; young and elderly subjects [169]; multiple input signals (both ECG and PPG) [18]; and the presence and absence of movement [171]. An assessment’s generalizability can be increased by using multiple datasets.

The methodology used to obtain reference BRs is highly important (see Section IV-A). If possible, reference BRs should be obtained independently from the input signals (ECG or PPG). For instance, ImP signals are often acquired using the same electrodes as the ECG. In contrast, gold standard spirometry signals are measured from air flow at the mouth (and nostrils) avoiding dependencies with input signals. Methods for estimating BRs from a reference signal should be carefully chosen and preferably evaluated. The reliability of manual breath annotations can be improved by using two independent annotators, particularly when signal periods containing disagreements between annotators are discarded [31]. Reliability can be assessed using interannotator agreement. When using an automated algorithm, its performance can be evaluated using manual annotations on a subset of the data [18].

#### 3) BR Algorithm(s)

The choice of BR algorithm(s) is straightforward in validation studies. The performance of one or a few algorithms is evaluated, without the need for additional comparator algorithms, to determine whether the proposed algorithm(s) perform sufficiently well for the chosen application.

There are additional considerations when choosing BR algorithms for exploratory studies. First, additional comparator algorithms should be included to contextualize the results, particularly if using a novel dataset since no comparative results will be available. Comparator algorithms should include leading algorithms from the literature (the Smart Fusion algorithm [8] is often used for this purpose [31], [106], [223], [226]). It may also be beneficial to include algorithms created by varying the technique used at a particular stage of the algorithm to identify techniques that improve performance [18], [226]. Second, the BR algorithms can be optimized in a preliminary analysis prior to assessment (ideally using a separate dataset). Aspects suitable for optimization include: window duration, whether or not to use a fusion technique [8], choice of beat detector, which respiratory signals to use, and the threshold for quality assessment [1]. For instance, the simulated dataset in [18] is suitable for verifying algorithm implementations. Third, the range of BRs that can be outputted by an algorithm should be fixed.

#### 4) Statistical Analysis

The nature of the statistical analysis differs between exploratory and validation studies. In exploratory studies, a wide range of statistics should be used to quantify different aspects of algorithm performance (such as errors, the proportion of windows for which a BR estimate is provided, power requirements, and the time delay between the start of signal acquisition and a BR estimate being outputted). The analysis need not identify the best algorithm. Rather, it should identify algorithm techniques that lead to improved performance. This may be aided by subgroup analyses of algorithms that use different techniques and of different subject populations. In validation studies, a primary statistic should be identified *a priori* with which to determine whether the algorithm performs sufficiently well. Ideally, a threshold value of this statistic, indicating sufficient performance, should be chosen *a priori* (such as a mean absolute error of < 2 bpm). Although there are no standardized performance thresholds, further guidance on selecting primary endpoints is provided in Section V-C. Additional statistics can also be used to quantify secondary aspects of algorithm performance.

The following should also be considered:

1) whether a statistical test is required to identify improved performance (such as the Wilcoxon signed rank test for paired data or the Wilcoxon rank sum test for unpaired data [34]);
2) the expected distribution of errors since parametric statistics such as LOAs are influenced more by nonnormal error distributions than nonparametric statistics such as coverage probability [18]; and
3) whether statistics are required to assess ability to detect apnoea [200], [203].

#### 5) Reproducibility

It is helpful to decide at the outset whether study resources will be made publicly available (including datasets, BR algorithms, and evaluation code) [18], particularly if ethical approval is needed. One should also decide whether the analysis should be reproducible [33].

## V Future Research Directions

### A Areas for Algorithm Development

There are several promising areas for BR algorithm development. First, little research has been conducted into the use of models of respiratory modulations in BR algorithms. Womack presented a model relating RSA to respiration [211]. If mathematical models such as this were incorporated into BR algorithms, then this could improve performance, particularly if they exploit relationships between the different respiratory modulations. Second, it has recently been proposed that the BRs provided by many different BR algorithms could be fused to improve performance [64], consequently reducing errors and increasing the proportion of windows for which a BR estimate is provided [226]. Further work is required to determine which BR algorithms should be used in this approach. Third, as the availability of annotated data increases, there is opportunity to use machine learning techniques in BR algorithms. Fourth, the utility of BR algorithms would be greatly enhanced if the uncertainty associated with a BR estimate was quantified since unreliable BR estimates could be easily discarded [13]. Fifth, further research is required to identify BR algorithms with low-computational requirements that are suitable for use in miniaturized devices such as wearable sensors [88], [221]. Finally, BR algorithms that use a breath detection technique could be used to estimate BR variability, which may have utility as a marker of mental state and disease progression [210].

### B Equipment

Research into BR algorithms has mostly used ECG and PPG signals acquired from routine equipment to assess the performance of algorithms. Some research has investigated alternative equipment for acquiring ECG and PPG signals, to either improve the performance of BR algorithms or to increase their utility.

Design considerations when using PPG signals include the following:

1) the anatomical site for PPG measurement (such as finger, ear, forehead, forearm, shoulder, wrist, and sternum), which may influence the strength of respiratory modulations [34], [68], [244]–[246];
2) the wavelength of emitted light [28], [247]; and
3) the use of transmission or reflection mode PPG [245].

Each of these factors may influence algorithm performance. Recent research indicates that low-fidelity PPG signals can be used with BR algorithms, such as those acquired at low sampling frequencies [34] or from smartphones or tablets [28], [29], [102], [115], [116]. This will potentially increase the utility of BR algorithms since they could be used in ubiquitous devices such as smartphones in resource-constrained settings [29], [102], [115], [116].

Design considerations when using ECG signals include: 1) whether suitable signals can be acquired without needing electrodes to be attached at several anatomical sites; and 2) whether multilead signals confer a significant benefit over single-lead signals. Klum *et al.* proposed that ECG electrodes positioned as little as 24 mm apart can be used to acquire respiratory signals [248]. This is promising for the implementation of BR algorithms in patch-style wearable sensors [249]. The use of textile-based systems to acquire ECG signals has also been investigated [174], [198], [215]. This could allow BR to be monitored by incorporating sensors into bed sheets [215] or a specialized t-shirt [128]. It has also been proposed that ECG signals could be acquired at locations other than the thorax such as the wrist [82] when an FM-based BR algorithm is used. Some studies have investigated the relative merits of single- and multilead ECG signals [197], [250] or fusion of respiratory signals acquired from single- and multilead signals [117]. It is not yet clear whether multilead signals provide improved performance, and therefore whether this should be considered when designing ECG acquisition equipment.

### C Applications

BR algorithms may have utility in a range of clinical and personal settings, with each setting having different requirements. The benefits and challenges of using algorithms in each setting are now described.

#### 1) Clinical Assessment

At present, BR is usually measured manually in clinical assessment in both hospitals and the community (as described in Section I-A). In contrast, BR algorithms could provide automated BR measurements. The key design challenges in this setting are to provide an accurate and precise BR, for most windows of input signal, preferably using the PPG since pulse oximeters can be attached quickly and easily, without any additional disposables. In particular, BR algorithm designs often include a tradeoff between performance and the proportion of windows for which BR estimates are provided [8]. The latter is likely to be more important since the present manual BR measurements have been reported to have poor performance (e.g., LoAs of −8.6 to 9.5 bpm [15]). An attractive case can be made for the use of BR algorithms in 4–6 hourly assessment of hospital inpatients since the time saved by using an automated method could reduce healthcare costs, and if BR algorithms provide improved performance, then this could improve patient safety.

#### 2) Clinical Monitoring Using Wearable Sensors

It is important that wearable sensors are capable of monitoring BR since BR is a sensitive marker of physiological deterioration preceding adverse events (see Section I-A). However, existing approaches for monitoring BR using wearable sensors are not ideal [18]. Many use impedance pneumography [251]. This is unobtrusive, involving measurement of variations in thoracic impedance with respiration through injection of a high frequency current into the thorax at ECG electrodes [252]. However, it is prone to errors caused by posture changes and motion [251], and has been observed to be imprecise (e.g., LoAs of −9.9 to 7.5 bpm [15] and −11.1 to 11.9 bpm [253]). Inductance plethysmography has also been used to monitor BR although this requires cumbersome chest bands [251], which may be too uncomfortable for prolonged monitoring [254]. More recently accelerometers have been used for BR monitoring [30] although this is still an ongoing area of research [255].

An alternative approach is to estimate BR from the ECG or PPG signals already acquired by many wearable sensors. This would allow BR to be monitored relatively unobtrusively, without an additional transducer. Wearable sensors can acquire ECG and PPG continuously, whereas clinical deterioration usually occurs over several hours. Therefore, one has the luxury of being able to discard data from which a BR cannot be confidently estimated. The key challenge is to provide accurate BR estimates since erroneous estimates may trigger false alerts, which are resource consuming and can erode trust in the wearable sensor [256]. Fusion techniques can reduce the frequency of erroneous estimates [18]. An additional challenge arises due to ambulatory data being highly susceptible to artifact, caused by poor sensor contact or movement [13], [198]. Methods for improving BR algorithms for use with wearable sensors include the following:

1) using SQIs to identify (and discard) artifactual data [231];
2) using techniques to reduce the influence of motion artifact [55], [88], [107], [135], [144], [198]; and
3) fusing BRs according to the uncertainties associated with each determined by either deriving features from extracted respiratory signals (such as variation in breath-to-breath intervals) [65] or analyzing the respiratory signals using Gaussian processes [13].

Furthermore, wearable sensors often acquire more than one signal from which BR could be estimated to improve robustness to artifact, such as ECG and PPG [44], [170] or ECG and accelerometry [65], [128]. The impact of motion on BR algorithms should be investigated further [59], as it is important both in clinical settings (such as during mobilization after surgery) and for fitness applications. Such a study would require reliable reference respiratory monitoring (such as spirometry) and would benefit from incremental increases in the level of motion (such as on a treadmill).

#### 3) Exercise Monitoring

It has been proposed that BR algorithms could be used in exercise monitoring. In healthcare, BR algorithms could be used during stress tests, which otherwise require a device (such as a spirometer), which is uncomfortable and may interfere with breathing [51]. Typically the ECG signal would be used since it is already monitored during exercise tests. This is a particularly challenging setting because: 1) input signals are greatly contaminated with motion artifact; and 2) ideally BR would be provided continuously, making it difficult to reject periods of low quality data. Temporal filtering has been widely used in this setting to increase BR algorithm performance [51], [53], [112]. In addition, multilead ECG signals have been used to improve performance, involving deriving cardiac rotation angles, including correction and rejection of outlying measurements [53].

BR algorithms could also be used in fitness devices. Many fitness trackers do not measure BR, but do acquire PPG for HR monitoring [31]. The ability to measure BR would be a valuable addition. This setting is less challenging than that of exercise tests since continuous BRs are not required, but could be provided only when expected to be reliable. In addition, BRs do not need to be as accurate as in healthcare. However, in this setting, the PPG is likely to be highly corrupted by motion artifact [13].

#### 4) Telemonitoring in the Home

Telemonitoring can be used to conduct frequent assessment of physiology in patients with chronic diseases living at home [257]. Telemonitoring setups often include a pulse oximeter, which patients use to assess their own HR and SpO_2_ [31]. However, it is difficult to measure BR remotely. A simple solution would be to incorporate a BR algorithm into a pulse oximeter. Indeed, Shah *et al.* recently found that BRs estimated from telemonitoring PPG data were predictive of exacerbations in chronic obstructive pulmonary (COPD) disease [258]. The design challenges in this setting are similar to those for clinical assessment although greater importance should be placed on obtaining an accurate BR than obtaining the measurement quickly. Ideally, a telemonitoring device would estimate BR in real time, continuing until an estimate with a high degree of confidence was available. The user would then be prompted to remove the device. Furthermore, if an abnormal BR was detected, then the user could be prompted to repeat the measurement to reduce the likelihood of false alerts.

#### 5) Remote Video Monitoring

The applications presented so far require sensors to be attached to the subject [259]. This requires manual intervention, may be poorly tolerated [260] and may cause discomfort and skin irritation [259], particularly when used over prolonged time periods. It has recently been proposed that BR algorithms could be applied to imaging-PPG signals acquired using noncontact video cameras [23]–[25], [27], [152]. Additional preprocessing steps are required to extract PPG signals from imaging-PPG videos for use with BR algorithms [23]: automatic detection of a region of interest (such as the face); synthesis of spatial information to extract a signal from the region; and color channel selection (using information from either a single or multiple color channels). HR and SpO_2_ can also be estimated from imaging-PPG signals, increasing their utility [25], [27]. Further work is required to determine whether BR measurements are best extracted from the cardiac-synchronous component of imaging-PPG signals, or from the changes in reflected light caused by the motion of breathing.

### D Translation Into Clinical Practice

Three key areas for future work to translate BR algorithms into clinical practice are now considered.

First, it is not clear whether different patient populations and clinical settings require different BR algorithms. This may arise due to differences in the requirements of algorithms (e.g., precision versus the proportion of windows for which an estimate is provided) or differences in respiratory physiology between patient groups (such as breathing patterns or the strength of respiratory modulations). Therefore, the first area for future work is to assess the performance of BR algorithms in the patient populations in which they are intended to be used. This will provide evidence for the expected performance of a BR algorithm in a particular target population (such as children [125], [189], [209]), and it will allow the most suitable BR algorithm for that population to be identified. For instance, Addison *et al.* have conducted several studies to assess the performance of BR algorithm performance across a range of populations (low-acuity hospitalized patients [43], [54] and patients in the postanesthesia care unit [151]) and in the presence of several pathophysiologies (respiratory disease [72], congestive heart failure [150], and COPD disease [149]). This provides an understanding of the performance of the Medtronic Nellcor BR algorithm (found to have LoAs of 0.07 ± 3.90 bpm in hospitalized patients [54]) and how its performance may be affected by pathophysiologies. Further, investigation of the impact of cardiac arrhythmias on performance is much needed [216] since arrhythmias may affect the physiological mechanisms responsible for respiratory modulation of the ECG and PPG [261].

Second, BR algorithms must be implemented in clinical monitors to be widely used in clinical practice. This review identified one clinical monitor in which a BR algorithm has been implemented: the Nellcor bedside patient monitoring system with reported LoAs of 2.25 ± 10.60 bpm when assessed against capnography derived BRs in patients undergoing procedural sedation and analgesia for endoscopy procedures [200]. This clinical implementation of a BR algorithm marks the beginning of a new phase in the use of BR algorithms since research into the potential clinical benefit of BR algorithms can be conducted with greater ease after clinical implementation. The process of implementing BR algorithms in monitors is likely to benefit from collaboration across multiple disciplines.

The third key area for future work is to conduct clinical trials to determine how BR algorithms can be used to deliver benefit to patients. These are likely to consist of two stages: an observational trial to determine whether a BR algorithm could be expected to be beneficial, followed by an interventional trial in which clinicians respond to the BRs, prompting changes in treatment. The first stage could be conducted using retrospective analysis of ECG or PPG signals, whereas the second is likely to require a clinical monitor in which a BR algorithm has been implemented. For example, Shah *et al.* used a PPG-based BR algorithm to perform a retrospective analysis of the utility of BR for prediction of exacerbations in COPD patients [258]. They observed that BRs derived from the PPG were predictive of exacerbations although the clinical utility of this approach needs to be assessed in an interventional trial.

### E Novel Physiological Insights

Novel insights into respiratory physiology can be gained by using BR algorithms in settings where it would otherwise not be practical to either measure BR or to monitor it continuously. In [195] and [262], an ECG-based BR algorithm was used to study changes in BR in the days following an acute myocardial infarction through secondary analysis of Holter ECG monitoring. This led to the insight that an elevated nocturnal BR (of ≥ 18.6 bpm) was associated with an increased risk of nonsudden cardiac death. It has also been suggested that BR algorithms can be used to investigate changes in breathing patterns due to pathology. In [185], an ECG-based BR algorithm was used to study changes in inspiration time, exhalation time, and the ratio of inspiration to exhalation time (as well as BR), associated with schizophrenia. In this particular study, no respiratory signal was available, so the BR algorithm provided additional insights.

## VI Conclusion

A wide range of algorithms to estimate BR from the ECG and PPG have been reported in the literature. These mostly conform to a standardized structure, with many different mathematical techniques proposed for each stage. BR algorithms are now being incorporated into clinical devices, with encouraging initial studies of their performance and utility in both hospitals and the community. Further work is required to identify the most suitable BR algorithms for use in different settings and to determine how BR algorithms can be used to deliver patient benefit. The great potential of BR algorithms is only likely to be realized through close collaboration between researchers, clinicians, and industrial partners.

## Supplementary Material

This paper has supplementary downloadable material available at http://ieeexplore.ieee.org, provided by the author. The material consists of data and code and additional details of the review methodology and results. Further information about the data and conditions of access can be found by emailing research.data@kcl.ac.uk.

## Figures and Tables

**Table I:** Filter-Based Techniques for Extraction of Respiratory Signals

Bandpass filter to eliminate frequencies outside the range of plausible respiratory frequencies [132]. Use (ensemble) empirical mode decomposition to extract a respiratory signal as either one particular oscillation mode (intrinsic mode function, IMF) or the sum of the IMFs indicative of respiration [170], [193]. Decompose signal using the discrete wavelet transform to reconstruct the detail signal at a predefined decomposition scale [59], optionally with automated selection of the mother wavelet [77]. Extract respiratory oscillation using principal component analysis (PCA) [144] or singular spectrum analysis [134] after identifying the periodicity using singular value decomposition. The use of PCA has been refined using multiscale PCA [137], and modified multiscale PCA [140], in which wavelet decomposition was combined with PCA. PCA has also been applied to intrinsic mode functions extracted using ensemble empirical mode decomposition [157], [158]. Extract the instantaneous amplitudes or frequencies of cardiac modulation using the continuous wavelet transform [41], the Teager-Kaiser energy operator [204], variable frequency complex demodulation [28], [67], the Hilbert transform [129], or the synchrosqueezing transform [79]. Filter using the centered correntropy function [90]. Decimate by detrending the signal, low-pass filtering to eliminate frequencies higher than respiration, and resampling at a reduced sampling frequency [85] of 1-2 Hz [10]. Extract an electromyogram signal from the high-frequency content (> 250 Hz) of the ECG caused by the activation of the diaphragm and intercostal muscles during respiration [95].

**Table II:** Feature-Based Techniques for Extraction of Respiratory Signals

Extract BW as the mean amplitude of peaks and preceding troughs [18] or the mean signal value between consecutive troughs [180]. Extract AM as the difference between the amplitudes of peaks and preceding troughs [8]. Extract FM as the time interval between consecutive peaks [8]. Extract peak amplitudes [8]. Extract trough amplitudes [180]. PCA of heartbeats [181], [227]. Extract the kurtosis between adjacent peaks [80]. Extract the morphological scale variation of part of the signal (e.g., QRS complexes) by comparing the current beat to a template beat [45]. Extract QRS durations [179]. Extract QRS areas [196]. Extract maximum Q–R or R–S slopes (using either a straight line fit [113] or fourth-order central moments [185]) or QRS-wave angles from the difference between them [113]. Extract PPG pulse widths [111]. Extract the difference between the durations of the upslope and downslope of the PPG [207]. Extract the direction or magnitude of the mean QRS vector axis using the arctangent of the ratio of QRS complex areas from two simultaneous ECG leads [195], [224]. Extract rotation angles of vectorcardiogram loops using multiple ECG leads [53]. Extract the main direction of the electric heart vector at a specific phase in the cardiac cycle (e.g., T-wave) [165]. Extract the pulse transit time using the R-wave of the ECG and the subsequent PPG pulse onset [44], [97], peak [213], or upslope midpoint [110]. Extract the maximum upslope during diastole of a venous signal extracted from dual wavelength PPG signals [219].

**Table III:** Techniques for Fusion of Respiratory Signals

Spectral averaging: calculate the individual power spectra of multiple respiratory signals, and then find the average spectrum [110], [114], [196]. Peak-conditioned spectral averaging [110], [114]: only sufficiently *peaked* spectra are included in calculation of a *peak-conditioned* average power spectrum. To qualify, a spectrum must have a certain proportion of its power within an interval surrounding the frequency corresponding to the maximum power [51] or the previous BR estimate [114]. Cross power spectral analysis: calculate the individual power spectra of multiple respiratory signals, and then multiply the spectra [117]. Cross time-frequency analysis [168]: use the smoothed pseudo Wigner–Ville distribution to estimate time–frequency spectra between two signals. Time–frequency coherence [168]: used to measure the degree of coupling between two signals. Vector autoregressive (AR) modeling [138]: the poles of multiple AR models (one for each respiratory signal) are calculated. Only those poles that are common to both models, and which fall within the range of plausible respiratory frequencies, are used to extract a respiratory signal. Point-by-point multiplication of signals [109]. Use of a neural network with multiple input signals to identify periods of inhalation and exhalation [32], [44].

**Table IV:** Techniques to Estimate BR from a Respiratory Signal

*Frequency-based techniques*
Spectral analysis: identify the respiratory frequency from a power spectrum calculated using either: the fast Fourier Transform [8] (which can operate on unevenly sampled signals [87]), AR spectral analysis using Burg or Yule-Walker algorithms [199], the Welch periodogram [111], the short-time Fourier transform [192], the Lomb-Scargle periodogram (which can operate on unevenly sampled signals) [52], or sparse signal reconstruction (which can be applied to multiple respiratory signals) [222], [223]. The BR is usually identified as the frequency corresponding to the maximum spectral power in the range of plausible respiratory frequencies although other approaches have been proposed [224]. Identify the respiratory frequency as the dominant frequency of a scalogram calculated using the continuous wavelet transform [41]. Identify the common frequency component in multiple respiratory signals using the weighted multisignal oscillator based least-mean-square algorithm [92]. Estimate instantaneous BR [92] using an adaptive notch filter [106], [173] or an adaptive bandpass filter [155]. Find periodicity using the autocorrelation function [184]. Estimate the instantaneous BR from either a single signal or multiple signals using a bank of notch filters [153], [154]. Autoregressive all-pole modeling, with BR estimated from the frequency of either the highest magnitude pole [10], or the lowest frequency pole [85]. The (order reduced) modified covariance method has also been used [136], [139] Use Gaussian process regression to estimate periodicity [177].
*Time-domain breath detection techniques*
Detect breaths using peak detection. Detect breaths by identifying zero-crossings with a positive gradient (after detrending) [32]. Detect breaths from peaks and troughs using (adaptive) thresholding to identify those breaths that have been reliably detected [74], [147], [184]

**Table V:** Methods Used to Assess BR Algorithm Performance

Category	No. publications (%)
Application of BR algorithms	
*Number of algorithms assessed*	
1	94 (48.0)
2–5	76 (38.8)
6–10	17 (8.7)
11–15	6(3.1)
≥ 16	3 (1.5)
*Input signal(s)*	
ECG	98 (50.0)
PPG	112(57.1)
Fusion of ECG and PPG	5 (2.6)
Pulse transit time	8 (4.1)
*Window duration [s]*	
< 30	10(5.1)
30–59	46 (23.5)
60–89	50 (25.5)
≥ 90	10(5.1)
Unknown	78 (39.8)
Datasets	
*Age(s) of subjects [years]*	
0–0.1: Neonate	5 (2.6)
0.1–17: Pediatric	27 (13.8)
18–39: Young adult	122 (62.2)
40–69: Middle-aged adult	76 (38.8)
≥ 70: Elderly adult	50 (25.5)
Unknown	57 (29.1)
*Level(s) of illness*	
Healthy	127 (64.8)
Sick in community	22(11.2)
Acutely ill	15 (7.7)
Critically ill	52 (26.5)
unknown	9 (4.6)
*Type(s) of breathing*	
Spontaneous	150 (76.5)
Metronome	45 (23.0)
Ventilated	32 (16.3)
Simulated	7 (3.6)
unknown	25 (12.8)
*Number of datasets used*	
1	164 (83.7)
2	30 (15.3)
3	1 (0.5)
4	1 (0.5)
Comparison with reference BRs	
*Reference BR equipment*	
Air flow or pressure	45 (23.0)
Impedance pneumography (ImP)	48 (24.5)
Capnography	33 (16.8)
Inductance plethymography (InP)	14(7.1)
Piezoelectric	9 (4.6)
Strain gauge	19 (9.7)
Metronome	9 (4.6)
Other	22(11.2)
None	5 (2.6)
unknown	26 (13.3)
*Common statistical measures*	
Error statistic	127 (64.8)
Breath detection statistic	19 (9.7)
Bias	46 (23.5)
Limits of agreement (LOAs)	46 (23.5)
Correlation	27 (13.8)
Proportion of windows	14(7.1)

**Table VI:** Publicly Available Datasets Used to Assess BR Algorithms

Dataset	Ref	No subjs	Age	ECG	PPG	Resp sigs	Type of breathing	Level of illness
Datasets containing breath annotations								
BIDMC	[31]	53	adult	Y	Y	ImP	spont, vent	critical
CapnoBase	[8]	42	paed, adult	Y	Y	CO_2_	spont, vent	surgery, anesthesia
Datasets without breath annotations								
MIMIC-II	[234]	23,180	paed, adult	Y	Y	ImP	spont, vent	critical
MGH/MF	[235]	250	paed, adult	Y	N	ImP, CO_2_	spont, vent	critical
MIMIC	[236]	72	unk	Y	Y	ImP	spont, vent	critical
VORTAL	[18]	57	adult	Y	Y	ImP, Press	spont	healthy
Fantasia	[237]	40	adult	Y	N	unk	spont	healthy
UCD Sleep Apnea	[238]	25	adult	Y	N	flow	spont	healthy, apnea
CEBS	[239]	20	adult	Y	N	piezo	spont	healthy
ECG and resp	[240]	20	adult	Y	N	flow, pleth	spont	healthy
MIT-BIH Polysomnographic	[241]	18	adult	Y	N	flow	spont, vent	healthy, apnea
Apnea-ECG	[242]	8	adult	Y	Y	InP, flow	spont	healthy, apnea
Portland State	[243]	1	paed	Y	Y	unk	unk	critical

Definitions: Age—pediatric (paed); respiratory signals (Resp Sigs)—capnometry (CO_2_), piezoresistive thoracic band (piezo), oral or nasal flow (flow), oral–nasal pressure (press), inductance plethysmography (InP), impedance pneumography (ImP), body plethysmography (pleth); Breathing—spontaneous (spont), ventilated (vent); unknown (unk).
